# Outcomes and Treatment of Lumbosacral Spinal Tuberculosis: A Retrospective Study of 53 Patients

**DOI:** 10.1371/journal.pone.0130185

**Published:** 2015-06-29

**Authors:** Tenglong Jiang, Jinming Zhao, Maolin He, Kun Wang, Mitra Fowdur, Yang Wu

**Affiliations:** 1 Division of Spinal Surgery, The First Affiliated Hospital of Guangxi Medical University, No.6 Shuang Yong Rd, Nanning, Guangxi, China; 2 Department of Orthopedic Trauma and Hand Surgery, The First Affiliated Hospital of Guangxi Medical University, No.6 Shuang Yong Rd, Nanning, Guangxi, China; 3 Guangxi Key Laboratory of Regenerative Medicine, Nanning, Guangxi, China; Fundació Institut d’Investigació en Ciències de la Salut Germans Trias i Pujol. Universitat Autònoma de Barcelona. CIBERES, SPAIN

## Abstract

**Study Strategy:**

A retrospective clinic study.

**Purpose:**

To evaluate the efficacy of conservative and surgical treatment for lumbosacral tuberculosis.

**Methods:**

This study retrospectively reviewed 53 patients with lumbosacral tuberculosis who were treated in our institution between January 2005 and January 2011. There were 29 males and 24 females with average ages of 37.53 ± 17.28 years (range 6–72 years). 11 patients were given only anti-TB drugs; the remainder underwent anterior debridement, interbody fusion with and without instrumentation, or one-stage anterior debridement combined with posterior instrumentation. Outcome data for these patients included neurologic status, lumbosacral angle, erythrocyte sedimentation rate value(ESR) and C-reactive protein value(CRP) were assessed before and after treatment.

**Results:**

The mean lumbosacral angles were 23.00°± 2.90°in the conservatively treated patients and 22.36°± 3.92o in the surgically treated patients. At the final follow-up, this had improved to 24.10o ± 2.96°in the conservatively treated patients and 28.13° ± 1.93°in the surgically treated patients (all P < 0.05). There were statistically significant differences before and after treatment in terms of ESR and CRP (all P < 0.05). All patients achieved bone fusion. The mean follow-up period was 32.34 ± 8.13 months (range 18 to 55 months). The neurological deficit did not worsen in any of the patients.

**Conclusions:**

It has been proven that conservative and surgical treatments are safe and effective and produce good clinical outcomes for patients with lumbosacral tuberculosis. The advantages of operation include thoroughness of debridement, decompression of the spinal cord, and adequate spinal stabilization.

## Introduction

Recently, due to HIV infection, bacterial resistance, and population migration, tuberculosis has become a leading cause of death in adults, especially in developing countries, with 1.4 million people dying of tuberculosis in 2011 [1]. Historically, the treatment for spinal tuberculosis has always been conservative and has included methods such as immobilization using body casts or plaster beds and a diet of nutritious food [2]. After anti-TB drugs became available for clinical use, many studies indicated that the administration of anti-TB drugs alone could effectively heal tuberculosis [3–6], but it may not be suitable in all situations, especially when treating patients with a risk of instability, progression of neurologic deficit, and failure of medical treatment. Many scholars have proposed that such patients should receive surgical treatment in order to prevent and correct spinal deformity, to improve neurological function, and to reconstruct spinal stability [7–9].

Thus far no consensus has been reached regarding the most effective means of managing spinal tuberculosis. Many studies have been conducted on thoracolumbar tuberculosis, whereas studies on the treatment for lumbosacral tuberculosis have been relatively rare. The incidence of spinal tuberculosis is approximately 2% of all cases of tuberculosis and lumbosacral tuberculosis accounts for 2–3% of spinal tuberculosis [10,11]. The presence of normal lordosis and the specific biomechanics of lumbosacral spine will influence the pattern of progression of the disease and the development of deformity [12]. This study was conducted to evaluate the efficacy of the two treatment methods for lumbosacral tuberculosis.

## Materials and Methods

### Ethics Statement

The study was approved by the Institutional Ethics Review Board at the First Affiliated Hospital of Guangxi Medical University; written informed consent was obtained from all patients or guardians.

### Patients’ general information

This study evaluated 53 patients with lumbosacral tuberculosis who were treated in our institution from January 2005 to January 2011 (Table 1). There were 29 males and 24 females with average ages of 37.53 ± 17.28 years (range 6–72 years), including 5 juveniles with average ages of 11.00 ± 5.10 years (range 6–17 years). All patients had symptoms of tuberculosis such as weight loss, moderate fever, and fatigue. Plain X-ray and MRI or CT were performed in all cases with the following results: vertebral bone destruction, uneven signals of bone, and smaller intervertebral space; 22 patients also showed a paravertebral abscess. 7 of the patients (13.2%) had hypertension, 4 (7.5%) had diabetes, and 9 (17.0%) had hepatitis B; each of these groups was subjected to periodical examination and related treatment. None of the patients were HIV positive. All patients received laboratory tests including complete blood count, ESR, and CRP. The diagnosis of tuberculosis was made with reference to symptoms, physical signs, and clinical and radiological findings, and was verified histopathologically after debridement in surgical patients. The neurological function assessed by the Frankel scoring system showed that 19 patients were grade B, 21 patients were grade C, and 9 patients were grade D.

**Table 1:** Summary of clinical data obtained in the 53 patients with lumbosacral spinal tuberculosis.

Methods	Case No.	Sex	Age(Y)	Neurologic status		Affected Segments	Lumbosacral angle(°)			Paravertebral Abscess
				BT	FU		BT	AT	FU	
ST	1	M	32	C	E	L5-S1	18.2	29.5	28.2	
	2	F	69	B	D	L4-L5	20.4	32.6	30.5	Yes
	3	F	56	C	D	L4-L5	21.1	28.1	27.6	Yes
	4	M	51	C	E	L4-L5	22.8	27.8	27.5	
	5	M	72	B	C	L5-S1	22.5	30.7	29.4	Yes
	6	F	70	D	D	L4-L5	30.2	31.1	30.1	Yes
	7	M	23	C	E	L4-L5	16.8	29.8	28.4	
	8	F	36	C	E	L4	19.2	29.2	28.2	Yes
	9	M	28	B	E	L4	20.4	28.3	28.0	
	10	M	33	C	E	L5-S1	16.2	29.3	29.2	Yes
	11	M	53	B	D	L4-L5	17.4	29.6	28.9	
	12	F	56	B	E	L4	23.1	29.5	28.2	
	13	F	21	B	E	L4-L5	18.5	31.9	29.2	Yes
	14	M	32	C	E	L5	21.7	27.7	27.5	
	15	M	28	B	E	L4-L5	26.4	30.4	28.8	
	16	M	33	C	E	L4-L5	24.5	28.5	27.8	Yes
	17	F	33	D	E	L4-L5	18.9	30.6	29.4	
	18	F	24	C	E	L5	22.3	30.8	30.3	
	19	M	65	B	D	L4	30.6	31.7	29.5	Yes
	20	M	43	C	E	L4	26.2	28.2	27.9	
	21	M	23	E	E	L4-L5	24.3	30.5	29.2	
	22	M	40	B	E	L4	22.5	28.9	28.5	
	23	F	28	C	E	L4-L5	27.5	32.2	29.9	Yes
	24	F	23	C	E	L5-S1	20.1	29.5	28.9	
	25	M	31	D	D	L4-L5	23.6	30.3	29.1	
	26	F	18	C	E	L4	18.5	28.7	27.2	
	27	F	32	C	E	L4-S1	24.5	29.1	28.1	Yes
	28	M	69	B	D	L4-L5	27.2	29.9	29.5	
	29	F	52	C	E	L4-L5	26.4	29.7	27.8	Yes
	30	M	58	B	D	L4-L5	19.8	28.4	27.8	
	31	F	68	B	C	L5-S1	17.1	27.6	27.2	Yes
	32	F	46	B	D	L4-L5	19.2	29.4	28.6	Yes
	33	M	28	B	E	L4-S1	21.2	29.0	28.5	
	34	F	34	C	E	L4	27.5	31.6	29.7	
	35	M	28	B	E	L4-L5	14.5	28.6	27.9	
	36	M	30	C	E	L5	25.5	29.7	29.0	Yes
	37	M	47	B	D	L4-S1	19.8	28.8	27.7	
	38	F	26	C	E	L5	20.3	29.9	29.2	Yes
	39	M	51	B	E	L4-L5	22.8	27.5	27.1	
CAC	40	M	31	C	E	L5-S1	18.3	20.5	21.3	
	41	F	58	B	D	L5-S1	24.5	27.7	28.1	Yes
	42	M	41	C	D	L4-L5	19.6	20.8	21.2	
	43	F	55	E	E	L4	22.1	22.9	22.4	
	44	F	24	D	E	L4-L5	25.2	26.8	27.9	Yes
	45	M	30	D	E	L5	23.1	24.9	23.8	
	46	M	33	E	E	L4-L5	20.8	21.2	20.5	
	47	F	52	B	D	L4-L5	23.5	22.7	23.7	
	48	F	20	D	E	L4-L5	28.3	29.8	28.8	Yes
ST	49	M	6	C	E	L5-S1	28.6	27.3	22.6	Yes
	50	F	8	D	E	L4-L5	25.3	26.9	22.3	
	51	M	8	D	E	L4	25.5	25.2	21.2	Yes
CAC	52	F	16	E	E	L4-L5	21.8	22.3	22.6	Yes
	53	M	17	D	E	L4	25.8	25.2	24.8	

This table shows Summary of clinical data obtained in the 53 patients with lumbosacral spinal tuberculosis. **ST**: surgical treatment, **CAC**: conservative anti-TB therapy; **BT**: before treatment, **AT**:6 months after treatment, **FU**: at the Final Follow-up.

### Inclusion criteria

Patients who were diagnosed with lumbosacral tuberculosis and had not previously received anti-TB therapy and debridement or radical depression surgery were included. The indications for surgery included severe back and/or radicular pain, a developing neurological deficit, significant kyphosis (> 30°), or progressive deformity [13,14].

### The method for conservative anti-TB therapy

Most lumbosacral tuberculosis patients could be effectively treated with medication, especially in the early stage of the disease [2–5]. The patients with a highly probable diagnosis who were not confirmed microbiologically or histopathologically but in whom the diagnosis was supported by typical radiographic or clinical features and without operative indications or with surgical contraindication received Isoniazid (5 mg/kg/day, 10 mg/kg in children), Rifampicin (10 mg/kg/day), Ethambutol (15 mg/kg/day), and Pyrazinamide (20 mg/kg/day, 25 mg/kg/day in children) as a standardized and effective anti-TB therapy, sustained for at least 4 months. This was followed by a two-drug anti-TB treatment (Rifampicin and Isoniazid) for 6 to 9 months or longer, until the toxic symptoms improved.

Bed rest was recommended for patients until clinical symptoms improved (as indicated by pain relief and neurological improvement). Mobilization was permitted at an average of 3 to 4 weeks, depending on the clinical response to treatment.

### Surgery program

Two weeks prior to surgery, all patients were treated with Isoniazid (5 mg/kg/day, 10 mg/kg in children), Rifampicin (10 mg/kg/day), Ethambutol (15 mg/kg/day) and Pyrazinamide (20 mg/kg/day). In addition, 20 patients who were undernourished (hemoglobin < 100 g/L, albumin < 30 g/L) received nutritional supplement treatments. Surgery was performed when the ESR had either decreased or not increased.

The anterior debridement, interbody fusion with instrumentation surgery was performed under general endotracheal anesthesia. Patients were operated on in the lateral position, taking a standard anterolateral approach to the spine via a retroperitoneal flank incision. After routine exposure, the necrotic materials within the disc and the vertebral bodies were removed using curettes and pituitary forceps. If the paravertebral abscess was large, drainage from a stab incision was necessary to identify the margin of the lesion foci. For adult patients, adequate debridement of all infected materials was performed. After radical resection of the involved spine, distraction was performed between adjacent normal vertebrae to correct the kyphosis. At the same time, the spinal defect was measured and allograft bone transplantation was used to reconstruct the anterior column defect. A locking plate-and-screw system of appropriate length was used. After careful hemostasis, normal saline was used for space irrigation to eliminate residual tuberculous tissue. Finally, streptomycin (1.0 g) was locally administered and a drainage tube was inserted before the incision was closed. The debrided tissue underwent histopathological examination. The anterior debridement combined with posterior instrumentation and a fusion procedure performed in these surgeries were the same as those performed in a study by Wang et al.[15].

With regard to the surgical methods used on children, it is important to keep the growth potential of the vertebral body as high as possible; thus, gentle debridement rather than radical debridement was performed. There were three children whose surgical procedure included anterior debridement only: one 6-year-old and two 8-year-olds.

After surgery, patients resumed treatment with oral anti-TB drugs (Isoniazid, Rifampicin, Ethambutol, and Pyrazinamide) for at least 4 months. Bed rest was recommended for all patients and all were provided with nutritional and supportive therapy. Patients were encouraged to change positions in bed during the first three days following surgery. The drainage tube was removed when the volume was less than 50 mL per 24 h. After one week, patients were encouraged to stand or walk with the effective support of braces.

### Recorded data

Follow-up care consisting of physical and radiographic examinations, ESR and CRP values, along with assessment of recovery of neurological functions, correction of deformity, and success of bone graft fusion. The lumbosacral angle was measured by drawing lines along the posterior border of S1 and the posterior border of the first normal vertebra above the level of the lesion[16]. The methods recommended by Moon et al. [8] to evaluate fusion status. Surgical complications were reviewed.

### Statistical analysis

SPSS for Windows (version 16.0; SPSS, Inc., Chicago, IL, USA) was used for data analysis, A paired *t*-test was used to compare the pre-treatment and post-treatment clinical data including the ESR, CRP, and lumbosacral angle. The discrepancy of the normal distribution was analyzed by a rank-sum test. The results are reported as mean ± standard deviation (SD). A P-value of less than 0.05 was considered statistically significant.

## Results

### Clinical symptoms

Of the conservatively managed patients, all but two experienced some relief from the toxic symptoms of tuberculosis after one month of treatment with anti-TB drugs. The two remaining patients from this group experienced relief after six months. However, in the surgical patients, the recovery period was significantly shortened, with patients experiencing relief from the toxic symptoms of tuberculosis at a mean time of 5.5 days postoperative.

### Neurological function

On the whole, the neurological deficit did not worsen in any of the patients. Of those conservatively managed patients, there were 5 in grade D who improved to grade E and 1 patient in grade B who improved to grade C. The surgically treated patients achieved obvious improvement: 8 patients in grade B improved to grade E, 9 patients in grade B improved to grade D, 2 patients in grade C improved to grade D, and 19 patient in grade C improved to grade E (including 2 of the children patients). Two patients remained in grade D at the final follow-up (Table 2).

**Table 2:** Neurologic recovery according to Frankel scoring system.

Preoperative Frankel grade	No. of cases	Frankel grade at last Follow-up				
		A	B	C	D	E
A	0	0	0	0	0	0
B	19(1*17^▪^1^♦^)	0	0	2(1*1^▪^)	9(9^▪^)	8(7^▪^1^♦^)
C	21(19^▪^2^♦^)	0	0	0	2(2^▪^)	19(17^▪^2^♦^)
D	9(6*3^★^)	0	0	0	2(1*1^★^)	7(5*2^★^)
E	4(2*2^#^)	0	0	0	0	4(2*2^#^)

This table shows neurologic recovery according to Frankel scoring system

* the conservative adult patients

# the conservative children patients

▪ the adult patients who underwent anterior debridement, interbody fusion with instrumentation

♦the adult patients who underwent one-stage anterior debridement combined posterior instrumentation

^★^the children patients who underwent anterior debridement only.

### Correction of deformity and fusion

**Fig 1:**
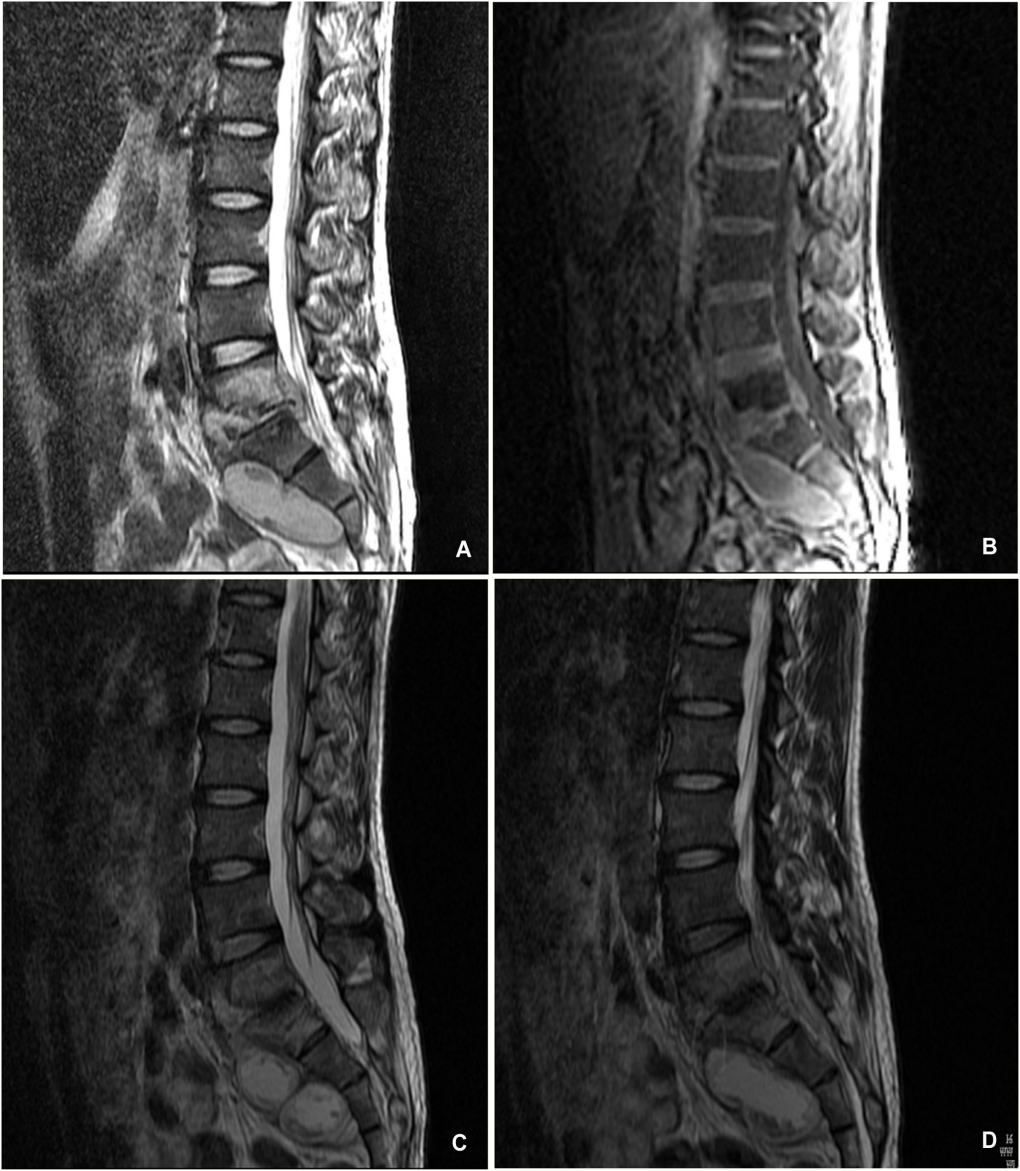
A 31-year-old man with lumbosacral spinal tuberculosis received conservative anti-TB therapy. (A,B)Pretreatment MRI shown destruction of vertebrae and paravertebral abscess with concomitant compression of the spinal cord.(C,D) Radiography taken at 15 months of follow-up showed no recurrence of tuberculosis.

The radiographic examination showed that interbody fusions occurred in all patients. In the conservatively managed patients, the average pretreatment lumbosacral angle was 23.00°± 2.90°(range 18.3°–28.3°), which improved to 24.10° ± 2.96° (range 20.5°-28.8°) at the final follow-up (P < 0.05). One adult patient, with the tuberculous foci at the L4–L5 and a large prevertebral abscess, received 10 months of anti-TB therapy and had healed with spontaneous interbody fusion and without residual kyphosis at the final follow-up (Fig 1). In the surgically treated patients, the lumbosacral angles showed a significant change from a preoperative mean of 22.36° ± 3.92° to a mean of 28.13° ± 1.93°at the last follow-up (P < 0.05).

### ESR and CRP

In the conservatively managed patients, the average pretreatment ESR and CRP were 27.91 ± 17.54 mm/h (range 3–67 mm/h) and 12.09 ± 4.06 mg/dL (range 4.4–19.8 mg/dL). These values decreased to 9.64 ± 2.91 mm/h (range 6–16 mm/h) and 1.52 ± 0.61 mg/dL (range 0.60–2.65 mg/dL) at the final follow-up (P < 0.05). In the surgically treated patients, the average preoperative ESR and CRP were 32.45 ± 17.78 mm/h (range 22–112 mm/h) and 18.61 ± 9.70 mg/dL (range 6.4–47.6 mg/dL), which decreased gradually and returned to normal levels 9 months after surgery. The average ESR was 4.24 ± 1.88 mm/h (range 3–12mm/h) and the average CRP was 0.95 ± 0.54 mg/dL (range 0.10–2.54 mg/dL) at the final follow-up (P < 0.05).

### Side effects and complications

All patients survived surgery. (Table 3). The average operation time was 137.45 ± 18.50 min (range 105–188 min) and the average blood loss was 287.62 ± 125.01ml(range 50–600 ml). Two patients had drug complications which resulted in abnormal liver function; one had this in combination with a gastrointestinal tract reaction, after adjusting the chemotherapy regimen, all patients gradually recovered. There were no injuries of the large vessels, cord, nerves, or ureter. Three patients had a transient neurological deficit but with complete neurological recovery at the 6 months follow-up. One case (2.4%) had leakage of the cerebrospinal fluid. After enforced recumbency and treatment with a large amount of low osmotic pressure fluid, the patient recovered after about 3 weeks. Three patients(7.1%) had hypokalemia and received intravenous rehydration to correct an electrolyte disorder. Three patients(7.1%) suffered persistent pain after operation and were treated with the administration of an analgesic. The pain disappeared one week postoperatively. Two patients developed superficial wound infections and were successfully treated by systemic antibiotic and debridement (Table 4).

**Table 3:** Recorded data and analysis of treatment.

Index	ST (n = 42)	CAC (n = 11)
**Surgery duration time (min)**	137.45 ± 18.50	-
**Blood loss (ml)**	287.62 ± 125.01	-
**NFIR (%)**	97.62%	54.55%
**ESR (mm/h)**		
**BT**	32.45 ± 17.78	27.91 ± 17.54
**AT**	10.40±4.01*	9.18±4.14*
**FU**	4.24±1.88* ^♦^	9.64±2.91* ^#^
**CRP (mg/L)**		
**BT**	18.61 ± 9.70	12.09 ± 4.06
**AT**	1.74 ± 2.77*	5.51± 4.06*
**FU**	0.95±0.54* ^#^	1.52±0.61* ^♦^
**Lumbosacral angle (°)**		
**BT**	22.36°± 3.92°	23.00°± 2.90°
**AT**	29.38°±1.52°*	24.07°±3.06°*
**FU**	28.13°±1.93°* ^♦^	24.10°±2.96°* ^#^
**LCA(°)**	-1.25°± 1.09°	0.03°± 0.80°

This table shows recorded data and analysis of treatment. **ST:** surgical treatment, **CAC:** conservative anti-TB therapy, **BT**: before treatment, **AT**:6 months after treatment, **FU**: at the Final Follow-up, **NFIR:** Neurological function improvement rate, **LCA:** Loss of correction angle.

* P < 0.05 vs. BT

# P > 0.05 vs. AT

♦ P < 0.05 VS. AT

Note: there were 4 conservatively managed patients in grade E

**Table 4:** Intraoperative and Postoperative complications.

Variables		AS	APS
**Intraoperative**	Large vessel injuries	0	0
	Ureter injuries	0	0
**Postoperative**	Wound infection	2	0
	Ileus	3	0
	Harvest site pain	2	1
	hypokalemia	2	1
	Leakage of cerebrospinal fluid	0	1

This table shows intraoperative and postoperative complications. **AS:** Anterior surgery; **APS:** Anterior-posterior surgery

### Follow-Up

A follow-up was conducted at the first 3 months, then every 3 months until the 12th month, then every 6 months up to 24 months after treatment, and thereafter, once a year to the final follow-up. The average follow-up period was 32.34 ± 8.13 months (range 18–55 months). Complete blood counts, ESR, liver function test, and serum uric acid levels were monitored to judge the efficacy of treatment and to monitor side effects, if any.

Comorbidities were subjected to periodical examination and related treatment. The surgical complications and their residuals were reduced and/or prevented by meticulous technique and good after-care. Of the 49 patients with a neurological deficit, 34 improved to grade E at the final follow-up. Of the 15 patients who did not improve to grade E, 11 patients improved to grade D, 2 patients improved to grade C and 2 patients had no change. CT scans showed that the spinal cord was decompressed sufficiently and had no dislocation, and all patients showed evidence of fusion. All patients became free of spinal tuberculosis without relapse, and the great majority of patients (51 patients, 96.2%) achieved a favorable status in which their abscesses had been absorbed or become smaller or calcified over a period of 3 to 12 months and disappeared at the last follow-up.

### Illustrative case

#### Case 1

The patient was a 31-year-old man who had been experiencing back pain and progressive weakness for 3 months. A neurologic examination revealed that the patient’s spinal cord function was a grade C according to the Frankel scoring system. A pretreatment MRI examination showed destruction of the L5–S1 vertebrae and compression of the spinal cord (Fig 1A and 1B). After one month of anti-TB drug therapy, the severe back pain was relieved. The patient continued standardized and effective therapy for 15 months. No recurrence of tuberculosis was noted in this patient during the follow-up period (Fig 1C and 1D).

#### Case 2

**Fig 2:**
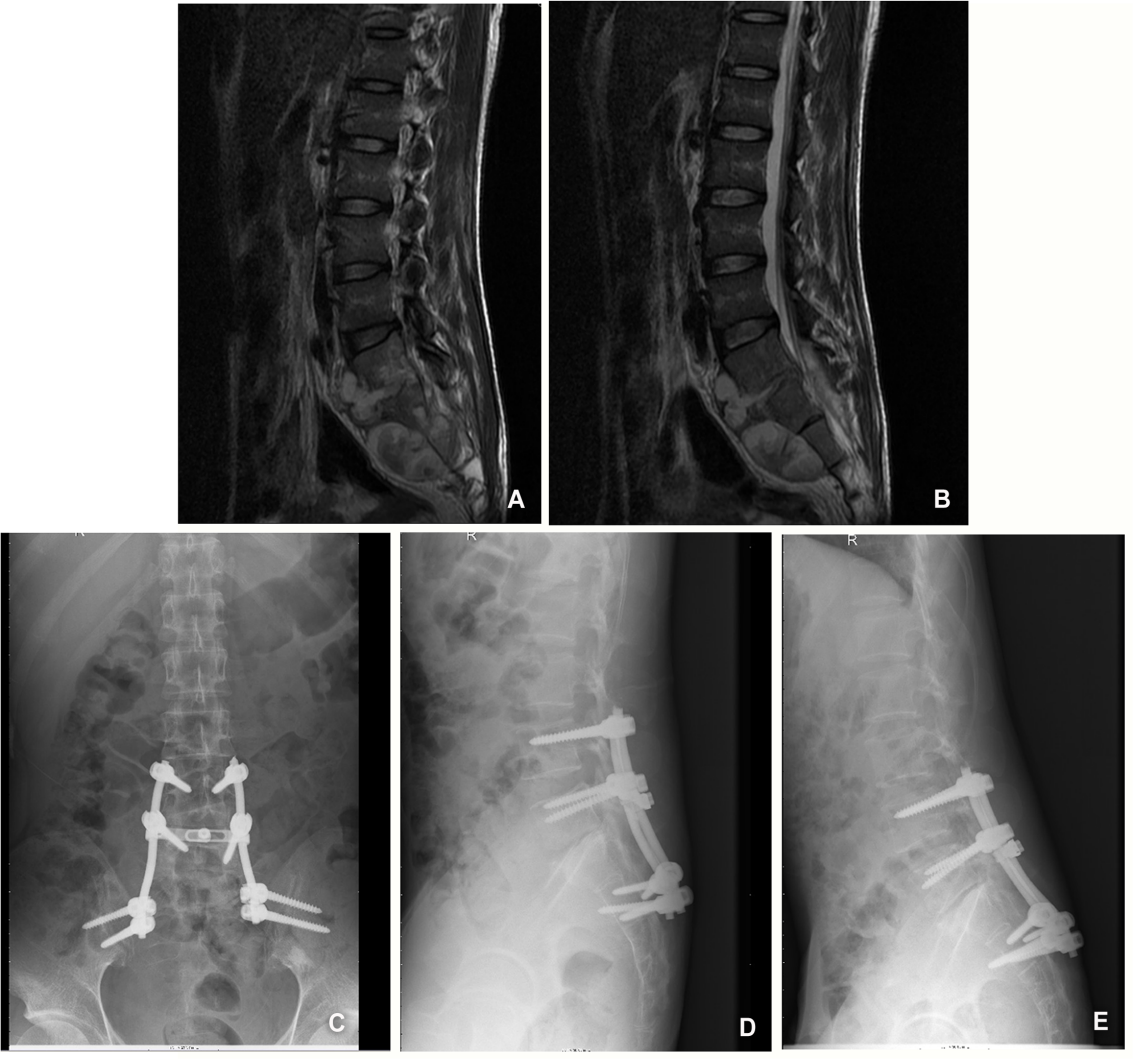
A 28-year-old man with lumbosacral spinal tuberculosis underwent anterior debridement, decompression combined with posterior plate fixation. (A,B)Pretreatment MRI shown destruction of the L4–S1 vertebrae and paravertebral abscess with concomitant compression of the spinal cord.(C) Immediate postoperative radiographs demonstrating anterolateral debridement, bone graft and internal fixation. (D,E) At 22 months’ follow-up, plain X-ray showed maintenance of the correction and solid fusion.

This 28-year-old man was admitted to the hospital for severe back pain and progressive weakness. A neurologic examination showed that the patient’s spinal cord function was a grade B according to the Frankel scoring system. Radiologic studies showed destruction of the L4-S1 vertebral bodies with anterior epidural and bilateral paraspinal abscesses and compression of the spinal cord (Fig 2A and 2B). The lumbosacral angle was 21.2° at the start of treatment. The patient was initially given oral anti-TB drug therapy for 2 weeks and then underwent anterior debridement and decompression combined with posterior plate fixation. The neurologic deficit improved immediately after surgery and with complete neurological recovery at the 6 months follow-up. The postoperative kyphosis correction was satisfactory and no significant loss of correction was identified at the final follow-up examination (Fig 2D and 2E).

#### Case 3

**Fig 3:**
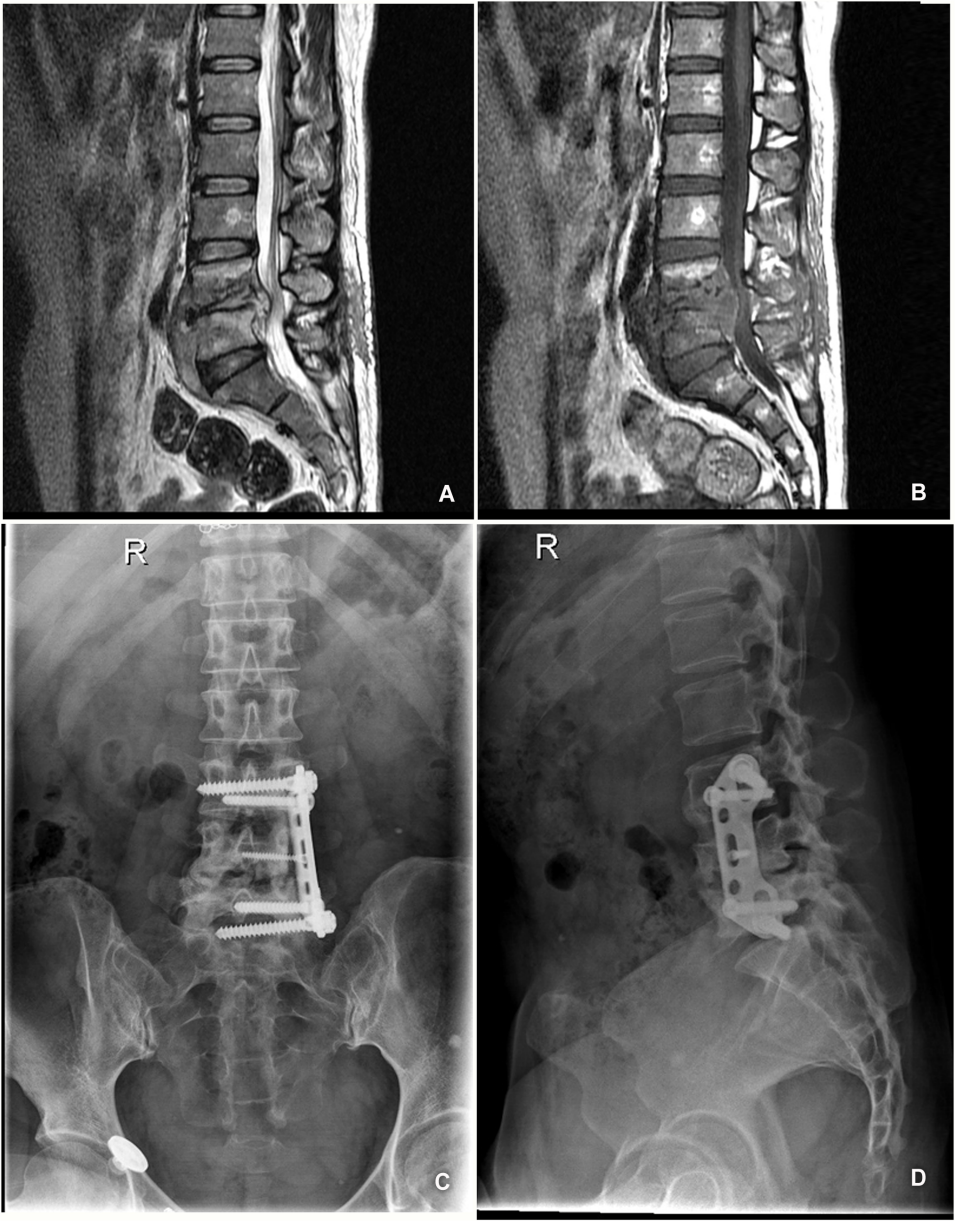
A 56-year-old woman with lumbosacral spinal tuberculosis received conservative anti-TB therapy. (A)Pretreatment MRI showed destruction of the L4–L5 vertebrae and formation of paravertebral abscess with concomitant compression of the spinal cord. (B) After 6-week anti-TB therapy, the back pain became more serious. (C,D) No recurrence of TB was noted in this patient during the follow-up period.

This patient was a 56-year-old woman who had been experiencing moderate back pain, stiffness, and progressive weakness of the extremities for 2 months. A neurologic examination revealed that the patient’s spinal cord function was a grade C according to the Frankel scoring system. Preoperative MRI studies showed destruction of the L4-L5 vertebrae and compression of the spinal cord (Fig 3A). After 6 weeks of anti-TB drug therapy, the back pain became more serious (Fig 3B). Then, the patient underwent an L4 corpectomy, decompression and anterior plate fixation. Pain relief was observed immediately after surgery and the patient regained full motor strength 3 months later. No recurrence of tuberculosis was noted in this patient during the follow-up period (Fig 3C and 3D).

## Discussion

Spinal tuberculosis has been around for a very long time. Despite its common occurrence and the high frequency of long-term morbidity, there are no straightforward guidelines for the diagnosis and treatment of spinal tuberculosis [17]. Most scholars agree that spinal tuberculosis is a “medical condition” [2,5,6,18]. Anti-TB drug treatment has played an important role in the treatment of tuberculosis, especially in the early infectious stage. Furthermore, various studies have shown that the majority of patients (82–95%) of spinal tuberculosis respond very well to medical treatment [17]. Konstam and Rajasekaran [11,19] have shown the positive effects of outpatient treatment with anti-TB therapy. Abhay Nene et al. [6] reported that over 98% of their patients (69 of 70) were successfully treated conservatively, without the need for surgical decompression. In our institution, the patients (including the 11 conservatively managed patients in the present study) with spinal TB who did not meet the surgical indication were treated conservatively as outpatients. Good healing can be achieved after anti-TB drug therapy.

The major shortcoming of conservative anti-TB therapy for spinal tuberculosis is the lengthy duration of drug treatment. Anti-TB drug therapy is typically continued for 9–18 months—or longer—until clinic symptoms have improved. Many patients find it very difficult to bear such a long period of treatment. In addition, long-term drug administration makes supervision laborious and time-consuming, and long-term combination therapy can easily lead to side effects, thus increasing the incidence of tissue and organ damage. In our study, 2 patients had drug complications. After adjusting the chemotherapy regimen, all patients gradually recovered. When patients are treated with anti-TB chemotherapy, we should enhance monitoring to avoid a serious adverse reaction.

Anti-TB drug therapy may not be appropriate in all situations, especially in cases where there is a risk of instability, progression of neurologic deficit, and failure of medical treatment. Spinal surgery involves removing the spinal cord lesion, relieving nerve compression, and reconstructing spinal stability, which effectively relieves spinal cord compression and prevents kyphosis. Early diagnosis and prompt treatment is necessary to prevent permanent neurological disability and to minimize spinal deformity [18,20].

With regard to the surgery, surgeons point out that “No patient with neurological deficit recovered or stabilized with non-operative management” and recommend that advanced neurological deficit be a surgical indication [5,11,21–23]. Various studies report different surgical indications; however, the current clinically recognized indications for surgery are: severe back and/or radicular pain resistant to conservative treatment, developing neurological deficit, significant kyphosis (> 30°), or progressive deformity [13,14].

As of now, several surgical approaches have been introduced, including anterior spinal fusion, anterior-posterior spinal fusion, posterior spinal fusion alone, and posterior spinal fusion followed by anterior spinal fusion. The surgical method used in any given case varies according to neurological deficits, spinal deformities, abscesses, and radicular or dural compression.

In 1960, Hodgson and Stock presented a thorough anterior release, because spinal tuberculosis mostly damaged the spinal anterior column [9]. Surgical treatment through an anterior approach was long considered to be the “gold standard” because an anterior radical surgical excision leads the surgeon directly to the lesion, provides optimal visualization, decompresses the spinal cord directly and completely, and prevents the possible progression of a kyphotic deformity [7,9,24,25]. In the present study, we performed an anterior debridement, interbody fusion with instrumentation surgery in 36 patients, the lumbosacral angle improved from the preoperative average of 22.04° ± 3.87° to a postoperative average of 28.60° ± 0.90° (P < 0.05) At the final follow-up examinations, of the patients with a neurological deficit, 24 showed complete neurological recovery.

Some authors have reported that it is necessary to add posterior stabilization to restore spinal stability and correct kyphotic deformity [26,27]. Furthermore, when the facet joints are gaping, there is vertebral slippage, and the progression of kyphosis can be observed at any examination time, posterior instrumented stabilization and fusion have been strongly recommended [5,8,28]. Wang et al. reported a 100% fusion rate after one-stage anterior debridement, bone grafting, and posterior instrumentation [15].

Five children participated in our study. The destruction of bone in children is more rapid and serious than that of adults. In children, because of the growth characteristic, ligamentous laxity, and poor muscle control, the kyphosis is always more serious. The mean lumbosacral angle in the 5 child patients was 21.5°±3.6° (range 18.6°-25.5°). In consideration of the growth process in children, during anterior focus debridement, it is important to retain the healthy bone and growth plate for maintenance of spinal growth after surgery. We think radical anterior surgery in children with spinal tuberculosis destroys the anterior growth and limits the capacity for spinal remodeling. Therefore, the child patients underwent gentle debridement and allograft bone transplantation. An external brace fixation was applied post operation.

It is important to understand the nature of spinal tuberculosis and its surgical indications, which are based on the level of lesions, severity of deformity, and neurology. In our study, patients received a thorough evaluation before undergoing surgery. The preoperative conditions should be investigated before entering the operating room to avoid preventable complications. We think that preventing and properly treating various complications are key factors for improving the success rates of surgery and to facilitate the recovery of patients. Finally, we found that during the follow-up period, there were no grafts or instrumentation-related stabilization problems. There were statistically significant differences before and after treatment in regards to lumbosacral angles, neurological status, ESR, and CRP. Positive clinical outcomes were achieved in both groups.

The main drawback of our study is its retrospective character. The limitations of our study include the small number of child patients, short observation time, and that some outpatients were lost to follow-up. In all the patients in this study, the disease was affecting fewer than three vertebrae. In addition, because lumbosacral spinal tuberculosis can produce severe deformity in children, which continues to grow even after the tuberculosis has healed, a long-term follow-up into adulthood for these patients is necessary.

## Conclusions

For the treatment of spinal tuberculosis, there is no acknowledged and definitive classification system. Our study shows that conservative anti-TB drug therapy and surgical treatment are safe and effective techniques with good clinical and radiological outcomes for patients with lumbosacral spinal tuberculosis, especially for those who are diagnosed early and who have fewer affected vertebrae. The advantages of surgery include thoroughness of debridement, decompression of the spinal cord, and adequate spinal stabilization.
